# Target-site mutations (AChE-G119S and *kdr*) in Guangxi *Anopheles sinensis* populations along the China-Vietnam border

**DOI:** 10.1186/s13071-019-3298-x

**Published:** 2019-02-07

**Authors:** Chan Yang, Xiangyang Feng, Nian Liu, Mei Li, Xinghui Qiu

**Affiliations:** 1 State Key Laboratory of Integrated Management of Pest Insects and Rodents, Institute of Zoology, Chinese Academy of Sciences, Beijing, 100101 China; 2 Guangxi Zhuang Autonomous Region Centre for Diseases Control and Prevention, Nanning, 530028 China; 3 University of Chinese Academy of Sciences, Beijing, 100049 China; 4 Institute of Physical Science and Information Technology, Anhui University, Hefei, China

**Keywords:** *Anopheles sinensis*, Haplotype, Guangxi Zhuang Autonomous Region, China-Vietnam border, G119S, Knockdown resistance (*kdr*), Acetylcholinesterase (AChE), Voltage-gated sodium channel (VGSC)

## Abstract

**Background:**

In South Asia, the epidemiology of malaria is complex, and transmission mainly occurs in remote areas near international borders. Vector control has been implemented as a key strategy in malaria prevention for decades. A rising threat to the efficacy of vector control efforts is the development of insecticide resistance, thus it is important to monitor the type and frequency of insecticide resistant alleles in the disease vectors such as *An. sinensis* along the China-Vietnam border. Such information is needed to synthesize effective malaria vector control strategies.

**Methods:**

A total of 208 adults of *An. sinensis*, collected from seven sites in southwest Guangxi along the China-Vietnam border, were inspected for the resistance-conferring G119S mutation in acetylcholinesterase (AChE) by PCR-RFLP (polymerase chain reaction restriction fragment length polymorphism) and *kdr* mutations in the voltage-gated sodium channel (VGSC) by sequencing. In addition, the evolutionary origin of *An. sinensis vgsc* gene haplotypes was analyzed using Network 5.0.

**Results:**

The frequencies of mutant 119S of AChE were between 0.61–0.85 in the seven *An. sinensis* populations. No susceptible homozygote (119GG) was detected in three of the seven sites (DXEC, LZSK and FCGDX). Very low frequencies of *kdr* (0.00–0.01) were detected in the seven populations, with most individuals being susceptible homozygote (1014LL). The 1014F mutation was detected only in the southeast part (FCGDX) at a low frequency of 0.03. The 1014S mutation was distributed in six of the seven populations with frequencies ranging from 0.04 to 0.08, but absent in JXXW. Diverse haplotypes of 1014L and 1014S were found in *An. sinensis* along the China-Vietnam border, while only one 1014F haplotype was detected in this study. Consistent with a previous report, resistant 1014S haplotypes did not have a single origin.

**Conclusions:**

The G119S mutation of AChE was present at high frequencies (0.61–0.85) in the *An. sinensis* populations along the China-Vietnam border, suggesting that the vector control authorities should be cautious when considering carbamates and organophosphates as chemicals for vector control. The low frequencies (0.00–0.11) of *kdr* in these populations suggest that pyrethroids remain suitable for use against *An. sinensis* in these regions.

## Background

Malaria is a deadly vector-borne disease in tropical and subtropical regions, with 216 million malaria cases and 445,000 deaths reported in 2016 worldwide [1]. In Vietnam, 14,941 confirmed malaria cases were recorded in 2014 [2], while neighboring China is on-track to eliminate malaria by 2020 [3]. One major obstacle to elimination is the importation of malaria parasites in infected travelers which was seen in returning workers from Africa and Southeast Asia [3]. Furthermore, frequent population movement across the China-Vietnam border is a factor that poses a risk of malaria transmission and re-emergence, particularly in the adjacent Province of Guangxi. Considering that vector control remains a key strategy in malaria prevention and that its efficacy is threatened by the increasing resistance of vectors to available insecticides, there is a need to assess the actual occurrence of insecticide resistance-associated genetic mutations in Guangxi *An. sinensis* along the China-Vietnam border.

Carbamate (CM) and organophosphorate (OP) insecticides target insect acetylcholinesterases (AChEs). These insecticides interfere in the normal neurotransmission of insects through inhibiting the activity of AChE [4–6]. Related studies have demonstrated that G119S substitution in AChE (AChE-G119S) is associated with insect resistance to OP and CM [7–11]. Recent surveys have revealed that the G119S occurs at high frequencies in many field populations of *An. sinensis* in Asia [12, 13].

Insect voltage-gated sodium channels (VGSC) are the targets of a variety of insecticides including pyrethroid (PY) and organochlorine (OC) insecticides [14–16]. It has been characterized that point mutations can reduce the sensitivity of VGSCs to insecticides, thus leading to insecticide resistance [15]. Several conserved insecticide resistance-related amino acid substitutions have been documented, such as leucine (L) to phenylalanine (F) at the 1014th amino acid of VGSC [17]. L1014F is the most common mutation in VGSC in anopheline mosquitoes in Africa, Asia and America. In addition to 1014F, other two mutations (1014S and 1014C) were detected in *An. sinensis* from Asia, including China [16, 18–20]. Interestingly, *kdr* frequencies in samples from western Guangxi of China were low, while those in samples from northeast Guangxi were high [20]. 1014S was also present in *An. sinensis* samples from southern Vietnam [21].

In this study, insecticide resistance conferring mutations in *ace-1* (encoding AChE) and *vgsc* genes were investigated in *An. sinensis* adult samples collected from seven sites in Guangxi along the China-Vietnam border. In addition, the possible evolutionary origin of *kdr* haplotypes was analyzed.

## Methods

**Fig. 1:**
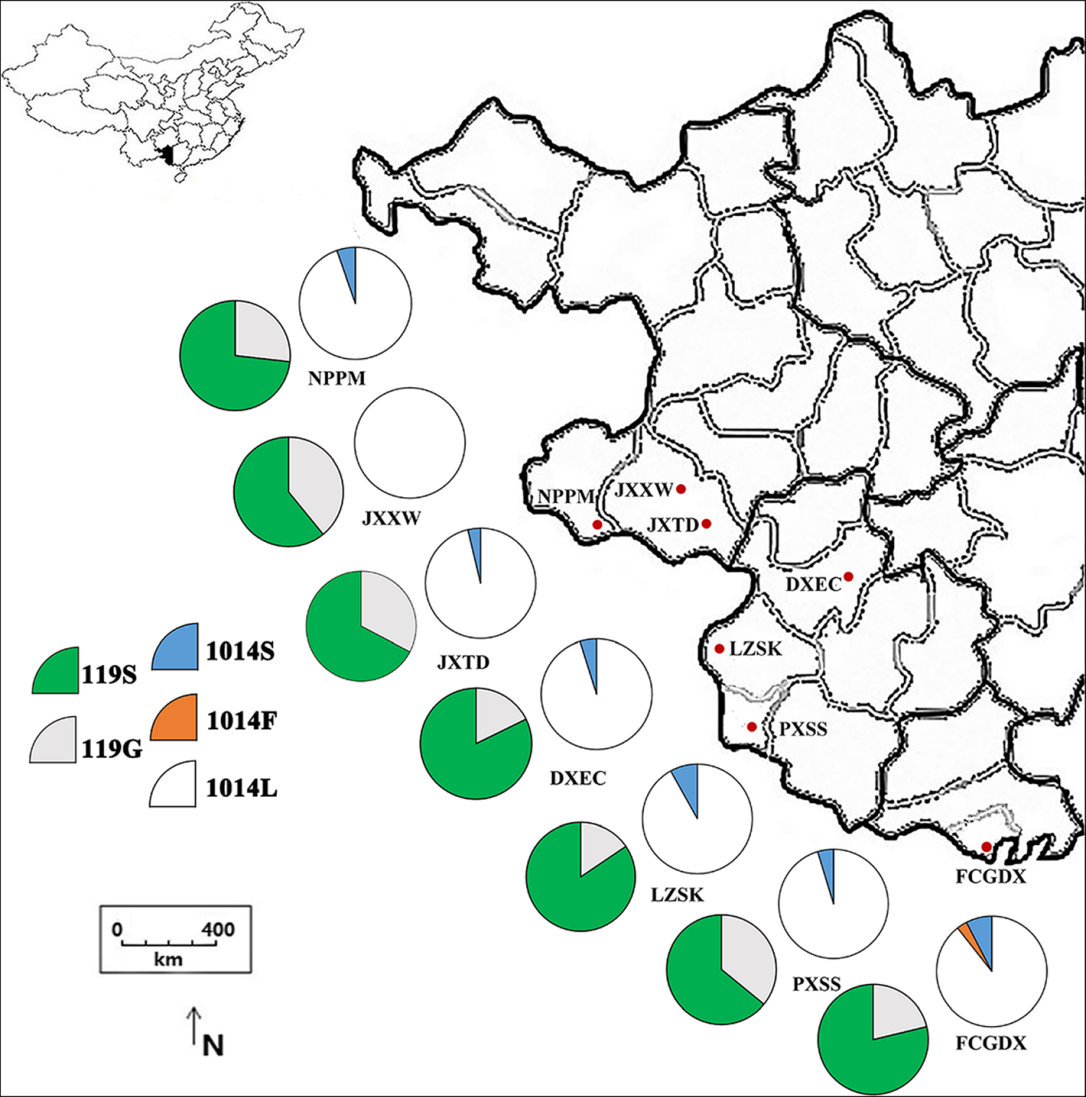
Distribution and frequency of *ace* and *kdr* alleles in *An. sinensis* populations along the China-Vietnam border. *Abbreviations*: NPPM, Pingmeng, Napo County; JXXW, Xinwei, Jingxi County; JXTD, Tongde, Jingxi County; DXEC, Encheng, Daxin County; LZSK, Shuikou, Longzhou County; PXSS, Shangshi, Pingxiang County; FCGDX, Dongxing town, Fangchenggang City

The seven sample-collecting sites were located in different villages of Guangxi near the Chinese-Vietnamese border (Fig. 1). Rice is the main crop planted in these villages. The large area of rice field provides an excellent environment for mosquito breeding. The local residents usually use mosquito nets and sometimes mosquito coils (containing S-bioallethrin or prallethrin) to prevent mosquito bites. The commonly used insecticides for rice pest control in these areas are diamides (e.g. chlorantraniliprole), neonicotinoids (e.g. imidacloprid) and organophosphorates (e.g. acephate and dimethoate).

*Anopheles sinensis* adults used in the study were caught by light trap (wave length 365 nm) between April 2015 and August 2017 at seven sites. At each sampling site, three houses were equipped with a light trap (one trap per house). The distance between houses was more than 50 m. The light trap was placed in the bedroom (1.5–2.0 m above the ground). The mosquitoes trapped from 19:00 h to 7:00 h for consecutive three days from each site were pooled, morphologically identified [22], and kept in 100% ethanol at 4 °C. Up to 33 *An. sinensis* were used for genotyping from each trapping effort.

The genomic DNA of individual mosquitoes was isolated according to the protocol described by Rinkevinch et al. [23]. Genotyping of codon 119 of *An. sinensis ace-1* gene was done by PCR-RFLP [12]. The frequency of the G119S mutation in each collection was recorded. Hardy-Weinberg equilibrium (HWE) was tested using the online software GENEPOP v.4.2 [24, 25].

**Fig. 2:**
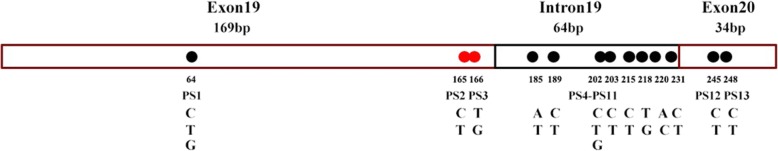
The nucleotide region of *An. sinensis vgsc* gene addressed in this study. Dots indicate the polymorphic sites (PS) in the obtained sequences. The red dots represent sites leading to nonsynonymous mutations. The positions of PS in the 255 bp sequence are numbered below the dots. The nucleotides for each PS are given

A fragment encompassing nucleotides corresponding to codon 1014 was amplified by PCR [20]. The PCR product from each individual was directly sequenced. Data from each sequencing was checked and cleaned manually. All confirmed DNA sequences were aligned using the Muscle programme in Mega v.6.0 [26], and polymorphic sites were identified (Fig. 2). The haplotypes of heterozygotes were clarified by T-A cloning (Transgen Biotech, Beijing, China) followed by clone sequencing (TSINGKE Biotech, Beijing, China). Network v.5.0 was used to analyze the evolutionary origin of *An. sinensis vgsc* haplotypes [27].

## Results

### The distribution and frequency of *ace-1* genotypes

The G119S allele was detected at frequencies ranging from 0.61 to 0.85 in the seven populations (Fig. 1). The three possible individual genotypes were observed, and all genotypes were detected to agree with Hardy-Weinberg equilibrium (Table 1). Notably, the susceptible homozygotes (119GG) were rare: no susceptible homozygote was detected at DXEC, LZSK and FCGDX, and the frequencies of 119GG were less than 0.10 at the other four sites (Table 1).

**Table 1:** Frequency of *Ace-1* genotypes in seven *An. sinensis* populations along the China-Vietnam border

Site	*n*	Frequency			Hardy-Weinberg equilibrium test (*P*-value)		
		GG	GS	SS	Probability test	Heterozygote excess	Heterozygote deficiency
NPPM	28	1	13	14	0.634	0.361	0.923
JXXW	23	2	14	7	0.377	0.212	0.957
JXTD	26	1	15	10	0.197	0.148	0.980
DXEC	31	0	11	20	0.553	0.342	1.000
LZSK	29	0	9	20	1.000	0.479	1.000
PXSS	32	2	19	11	0.148	0.119	0.978
FCGDX	33	0	14	19	0.300	0.175	1.000

*Abbreviations*: *NPPM* Pingmeng, Napo County, *JXXW* Xinwei, Jingxi County, *JXTD* Tongde, Jingxi County, *DXEC* Encheng, Daxin County, *LZSK* Shuikou, Longzhou County, *PXSS* Shangshi, Pingxiang County, *FCGDX* Dongxing town, Fangchenggang City

### Sequence polymorphisms of *An. sinensis vgsc* gene

Thirteen nucleotide polymorphic sites (PS) were identified from the 255 bp DNA fragments individually amplified from a total of 208 mosquitoes (Fig. 2). The 1st to 3rd PSs were located on exon 19, the 4th to 11th PSs on intron 19, and the 12th and 13th PSs on exon 20. The polymorphisms in 2nd and 3rd PSs resulted in amino acid substitutions (L/F/S) at codon 1014, and the nucleotide variations in 1st, 12th and 13th PSs represented synonymous mutations (Fig. 2).

### The distribution and frequency of *kdr* genotypes

Three *kdr* genotypes (1014LL, 1014LF and 1014LS) were identified from the samples (Table 2). The frequencies of the susceptible homozygotes (1014LL) ranged from 0.79 (FCGDX) to 1.00 (JXXW). The resistant heterozygote 1014LS was detected at six sites at frequencies ranging from 0.07 to 0.16, while the other resistant heterozygote 1014LF was found only at FCGDX at a frequency of 0.06 (Fig. 1). No significant deviation from HWE for the 1014 genotypes was observed in all the seven populations (Table 2).

**Table 2:** Frequency of *kdr* genotypes in seven *An. sinensis* populations along the China-Vietnam border

Site	*n*	Frequency			Hardy-Weinberg equilibrium test (*P*-value)		
		LL	LF	LS	Probability test	Heterozygote excess	Heterozygote deficiency
NPPM	28	25	0	3	1.000	0.947	1.000
JXXW	26	26	0	0	–	–	–
JXTD	27	25	0	2	1.000	0.981	1.000
DXEC	31	28	0	3	1.000	0.952	1.000
LZSK	31	26	0	5	1.000	0.839	1.000
PXSS	32	29	0	3	1.000	0.949	1.000
FCGDX	33	26	2	5	1.000	0.708	1.000

*Abbreviations*: *NPPM* Pingmeng, Napo County, *JXXW* Xinwei, Jingxi County, *JXTD* Tongde, Jingxi County, *DXEC* Encheng, Daxin County, *LZSK* Shuikou, Longzhou County, *PXSS* Shangshi, Pingxiang County, *FCGDX* Dongxing town, Fangchenggang City

### Diversity and frequency of *kdr* haplotypes

Eighteen *kdr* haplotypes were identified from the 208 *An. sinensis* individuals (Table 3). Among them, six haplotypes (i.e. 1014L15, 1014L16, 1014L17, 1014L18, 1014S5 and 1014S6) were new records.

**Table 3:** *kdr* haplotypes and their frequencies in seven *An. sinensis* populations along the China-Vietnam border

Haplotype	Polymorphic sites	GenBank ID	Frequency						
			NPPM	JXXW	JXTD	DXEC	LZSK	PXSS	FCGDX
1014L1	CTGACGCTGCTCC	KY014584.1	0.214	0.173	0.333	0.274	0.194	0.156	0.273
1014L2	CTGACGCCGCCTC	KY014585.1	0.482	0.519	0.389	0.435	0.210	0.313	0.242
1014L3	CTGACTCCGCCTC	KY014586.1	0.089	0.192	0.111	0.081	0.355	0.250	0.061
1014L4	CTGACTCTGCCTC	KY014587.1	0.018			0.048	0.016	0.063	0.045
1014L5	GTGACGCCGCCTC	KY014588.1	0.018	0.019	0.019		0.032	0.078	0.015
1014L6	CTGTCGCCGCCTC	KY014589.1	0.036	0.058	0.037	0.016	0.016	0.016	0.076
1014L7	CTGACGCTGCCCC	KY014590.1				0.016	0.016	0.016	
1014L8	CTGATGCCGCCTC	KY014591.1	0.054	0.019	0.037	0.048	0.048	0.047	0.152
1014L10	CTGACGCTGCCTC	KP763768.1	0.018	0.019		0.016			
1014L15^a^	CTGACGCTGATCC	This study			0.019	0.016	0.016	0.016	0.015
1014L16^a^	CTGACTTCGCCTC	This study			0.019		0.016		
1014L17^a^	TTGACGCTGCCCT	This study							0.015
1014L18^a^	CTGACGCCTCCTC	This study	0.018						
1014F1	CTTACGCTGCTCC	KY014598.1							0.030
1014S2	CCGACGCCGCCTC	KY014594.1	0.036		0.037	0.032	0.081	0.047	0.045
1014S3	CCGACTCCGCCTC	KY014595.1	0.018			0.016			
1014S5^a^	CCGACGCTGCTCC	This study							0.015
1014S6^a^	CCGTCGCCGCCTC	This study							0.015

Haplotypes with a GenBank number were previously described in Yang et al. [20]

^a^Newly identified haplotypes

The geographical distribution of *kdr* haplotypes was varied in the *An. sinensis* populations along the China-Vietnam border (Table 3). Seven (JXXW) to 13 (FCGDG) haplotypes were detected within these populations. Of the 13 susceptible haplotypes, 1014L1, 1014L2, 1014L3, 1014L6 and 1014L8 were widely distributed at the seven sites, and 1014L1, 1014L2 and 1014L3 represented the most prevalent haplotypes. Interestingly, the newly identified susceptible haplotypes, 1014L17 and 1014L18, were uniquely distributed at FCGDX and NPPM, respectively, at a low frequency (0.02).

Four 1014S (1014S2, 1014S3, 1014S5 and 1014S6) haplotypes were identified in this study (Table 3). The haplotype 1014S2 had higher frequencies and was more widely distributed than other 1014S haplotypes. The two newly identified 1014S haplotypes (1014S5 and 1014S6) and 1014F1 were only detected at FCGDX.

### Evolutionary origin of 1014S haplotypes

**Fig. 3:**
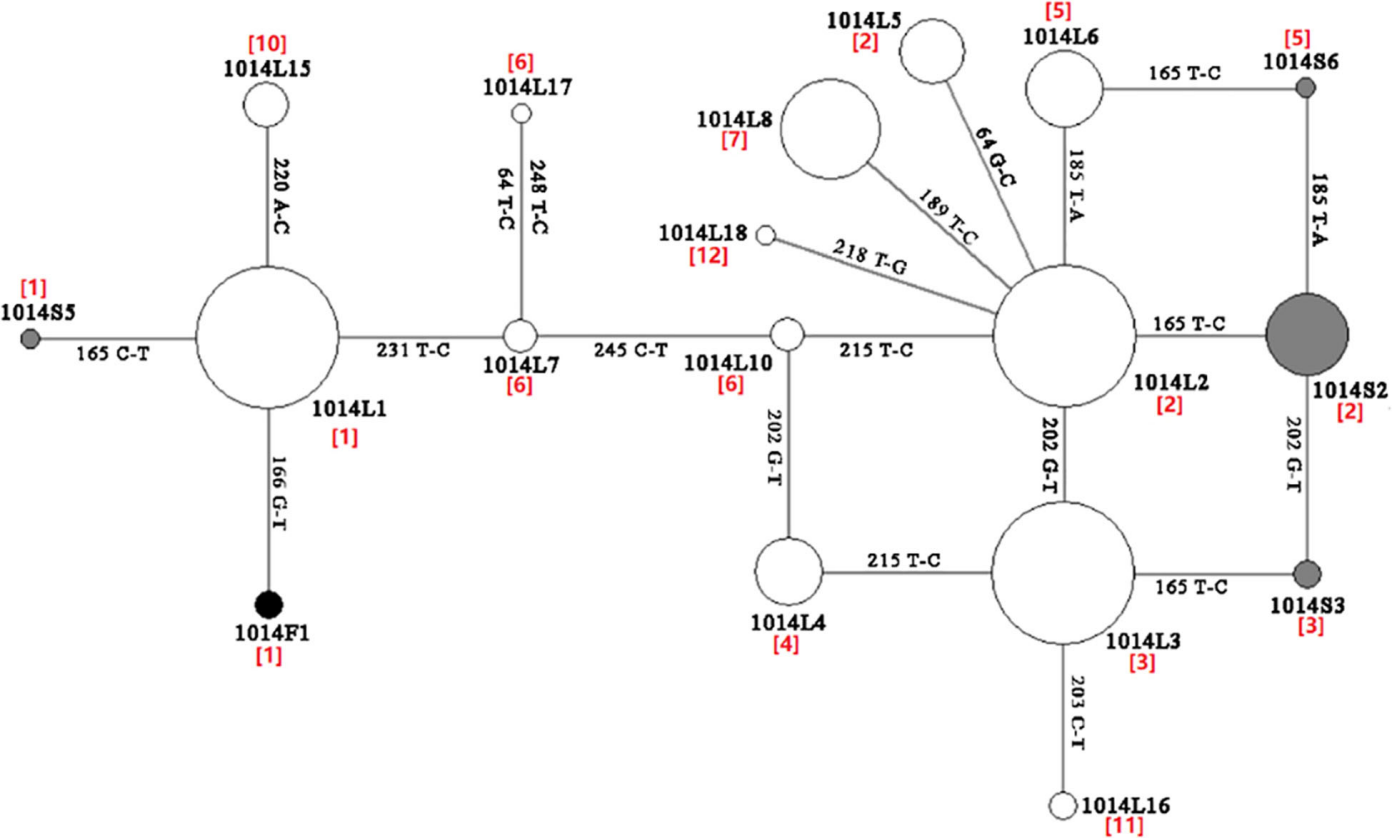
The network of *kdr* haplotypes identified in *An. sinensis* populations along the China-Vietnam border. White solid, black and grey circles represent 1014L, 1014F and 1014S haplotypes, respectively. The size of each circle is proportional to its corresponding frequencies. The number in brackets represents intron type

Network analysis showed that 1014S2, 1014S3 and 1014S6 were derived from 1014L2, 1014L3 and 1014L6, respectively, while both 1014S5 and 1014F1 evolved from 1014L1 through only one mutational step (Fig. 3). Notably, both 1014F1 and 1014S5 were detected only at FCGDX (Table 3).

## Discussion

For all seven field populations of *An. sinensis* collected in Guangxi along the China-Vietnam border, the frequencies of the resistant 119S allele were high. This result supports the published literature indicating that the G119S mutation is widely distributed in Guangxi [12]. The high frequency of the 119S allele indicates a strong risk of resistance to OP and CM in these regions (unfortunately no OP or CM bioassay data are available to test this indication). Historically, OPs have been used for pest control in China since 1950s and, as such, the high occurrence of the G119S may be a consequence of the long-term use of OP in agriculture. The observations that all *ace-1* genotypes did not deviate from HWE (Table 1) and that the resistant allele was present at a high frequency within the seven populations imply that mosquitoes carrying the G119S mutation may suffer no fitness cost under current natural conditions, or the cost mediated by G119S substitution may possibly be offset by unknown fitness modifiers.

Two different *kdr* mutations (1014S and 1014F) were identified in this study (Table 2, Fig. 1). These insecticide resistance-associated mutations occurred in heterozygous forms and at very low frequencies in these areas. Interestingly, the 1014C mutation, which is widespread and present at relatively high frequencies in northeast Guangxi [20], was not detected in this survey. In addition, 1014F1, which is widely distributed in several provinces of China including Guangxi [20], was detected only at FCGDX. By contrast, the 1014S allele was widely distributed along the border. The distinct distribution of the 1014S allele is likely a consequence of independent mutational events in different geographical locations. The distribution pattern observed in this study is consistent with previous observations showing that the frequency of *kdr* mutations decreases towards south and west from northeast [18–20, 28–30].

The present study demonstrates the geographical heterogeneities of *kdr* haplotypes and the presence of location-specific haplotypes (Table 3). For example, the haplotypes 1014S5 and 1014S6 were only detected in FCGDX. Furthermore, the genealogical analysis of *vgsc* haplotypes suggests that the 1014S2, 1014S3 and 1014S6 may have evolved from 1014L2, 1014L3 and 1014L6, respectively, adding support to the hypothesis that *kdr* mutations do not have a single origin [20]. Multiple origins of *kdr* mutations have also been documented in several other insect species [31, 32].

In the remote villages studied, no regular vector control programme has been implemented. The lack of pyrethroid selection pressure on the mosquito populations may explain why all *vgsc* alleles exhibit HWE, and why *kdr* is rare (Table 2). A recent preliminary survey indicated no loss of susceptibility to deltamethrin in *An. sinensis* adults collected from sampling sites same as in this study (conducted in June to August 2018 using the contact bioassay protocol recommended by China CDC, Dr. Fengxia Meng; personal communications). Based on the findings in this study, we suggest that pyrethroids remain suitable for use against *An. sinensis*. Noting that a strong insecticide resistance management programme should be implemented to maintain the susceptibility of *vgsc* alleles, it is recommended that the application of pyrethroids should not be taken as the sole measure for vector control and should be used in rotation or alongside insecticides with alternative modes of action.

## Conclusions

High frequencies (0.61–0.85) of the G119S mutation in AChE and low frequencies of *kdr* mutations (0.00–0.11) were detected in *An. sinensis* populations along the China-Vietnam border. 1014S was the most common *kdr* mutation in these areas. Network analysis revealed that the 1014S mutation did not have a single origin. The data suggest that the vector control authorities should be cautious when considering carbamates and organophosphates as control agents. Instead, pyrethroids are suitable for *An. sinensis* control in these regions.

## Abbreviations

AChE: Acetylcholinesterase
CM: Carbamate insecticides
Guangxi: Guangxi Zhuang Autonomous Region, China
HWE: Hardy-Weinberg equilibrium
*kdr*: Knockdown resistance
OC: Organochlorine insecticides
OP: Organophosphorus insecticides
PCR: Polymerase chain reaction
PY: Pyrethroid insecticides
RFLP: Restriction fragment length polymorphism
VGSC: Voltage-gated sodium channel
